# Preliminary Training for Probationers

**Published:** 1907-05-25

**Authors:** 


### Nursing Administration.

### PRELIMINARY TRAINING FOR PROBATIONERS.

The fact that the best working years of the nurse are over soon after 40 is a factor in determining income, which is the more serious from the age at which the nurse is able to begin her training. Can nothing be done to advance the time? Is there no part of the nurse's training which can be carried out before she enters hospital? To the first question the more strongly it is urged the more decidedly do the best authorities give a negative reply. No ordinary girl, and hospitals cannot deal with exceptions, is fitted before she is 22 or 23 to undertake the responsibilities which fall to the lot even of the probationer. With regard to the second question, there is more diversity of opinion. The desirability of some practical preparation of the probationer for the duties she will be called upon to perform is recognised in several hospitals, and in two at least—the London Hospital and Guy's Hospital—a preliminary course in a separate training school attached to the hospital is enjoined on all probationers. The drawbacks to this system are chiefly financial. It involves the maintenance of a separate building and staff, and the charge, which cannot exceed a guinea a week, is insufficient to defray the entire expenses of board, lodging, and instruction. For this reason alone it is improbable that the system of preliminary pupil schools will ever become general. Moreover, the charge, insufficient as it may be to defray the expense of the school, is more than many pupils can bear, and thus the choice of probationers is limited to those who can pay. Finally, it may be contended that the preliminary training afforded in a period of six weeks or three months does not go far enough. Too many subjects are crowded into the time, and yet to make the course longer is not only to increase the financial difficulty, but to run the risk of spoiling the freshness with which the probationer ought to attack her term of training, and the loss of which is ill-compensated by familiarity with routine. Is it necessary to subject the pupil to the expense and inconvenience of residence in order to impart the instruction which is the recognised preparation for a probationer's duties? The system practised at the Glasgow Royal Infirmary, where a complete course of training is given to intending probationers before they enter the wards, is in our opinion greatly to be preferred, the pupils either living at home or making their own arrangements for board and lodging during the continuance of the classes. In London it would require but little organisation to provide preliminary classes for all intending probationers, and the advantages which would be afforded without expense or trouble to nursing in general are readily apprehended. There is little doubt that the County Council would cordially co-operate in any scheme which had the approval of the hospitals, and would furnish at one or two convenient centres classes for intending nurses at minimum fees. Already under the Science and Art Department there are courses of instruction in physiology, anatomy, and hygiene which are too much ignored by hospital authorities. It remains to supplement them by courses arranged with a view to the manual work which the first year probationer ought to be skilled to perform on her first entry into the wards. The elementary work of sweeping, dusting, and cleaning; the care of bedding, and the special making of beds; the use and care of the thermometer; bandaging and the preparation of bandages and dressings; the making of sponges and pads, and the padding of splints; principles and practice in cleansing instruments, and generally in keeping metal work in good order. These are only a few of the practical details which should be familiar to the pupil, and in which considerable practice is needed before skill is attained. In addition to this practical course, should not the intending probationer be advised to go through a course of lessons in sick-room cookery and in massage or dispensing? It is hard upon a nurse, after three or four years' training, to be compelled to lose time before commencing her career, in learning things which she might quite suitably have been taught while she was waiting for admission to the training school. It is only because before entering hospital she was not alive to the importance of a knowledge of massage or dispensing that she omitted to acquire these arts, the possession of one or other of which may make all the difference in her ability to obtain some coveted post. Either branch of the work can be readily mastered by a girl of 18, and it is needless to say that such subjects as hygiene and anatomy are far more easy of assimilation by pupils able to devote some serious leisure to their study than in the hurry and worry of hospital life.

Two possible drawbacks suggest themselves to the preliminary instruction which we should like to see imposed upon all probationers before entering any hospital. The first is that wrong methods, difficult to eradicate, might be imparted to the pupils. This, however, if the classes were held under the direct sanction of the training schools, could not occur. In fact, the instruction given by an expert lecturer would be far above the level of that usually available for the unlucky probationer in her first few months, whose mission is to get in everyone's way, and find herself perpetually blamed for ignorance of things it has been no one's duty to teach her. Secondly, it will be urged that by means of such classes a new terror in the shape of an amateur nurse would be set loose upon the public. To guard against this danger, it would certainly be necessary to exclude from the instruction given any reference whatever to the treatment of disease. Medical and surgical nursing, the use of technical, medical, and surgical appliances, must be rigidly reserved for the time when practice can be wedded to theory. It is in the theoretical teaching of such subjects alone that danger lurks. It can do even the most blundering amateur no harm to be able to name her own bones, roll a bandage, and make a bed.

